# Supplementary material for: Dynalign II: common secondary structure prediction for RNA homologs with domain insertions
Source: Nucleic Acids Res. 2014 Nov 21;42(22):13939–48. doi: 10.1093/nar/gku1172 (PMC4267632; doi:10.1093/nar/gku1172)
Supplement: SUPPLEMENTARY DATA [file supp_gku1172_nar-02021-z-2014-File012.zip › manual/javadoc/comments/InterfaceSourceMenus.html]

A package that holds all menus used in the Java RNAstructure interface.
